# Association between Metformin Use and Cancer Stage at Diagnosis among Elderly Medicare Beneficiaries with Preexisting Type 2 Diabetes Mellitus and Incident Prostate Cancer

**DOI:** 10.1155/2016/2656814

**Published:** 2016-07-31

**Authors:** Amit D. Raval, Malcolm D. Mattes, Suresh Madhavan, Xiaoyun Pan, Wenhui Wei, Usha Sambamoorthi

**Affiliations:** ^1^Department of Pharmaceutical Systems and Policy, School of Pharmacy, West Virginia University, Morgantown, WV 26505, USA; ^2^Healthcore, Inc., Wilmington, DE 19801, USA; ^3^Department of Radiation Oncology, School of Medicine, West Virginia University, Morgantown, WV 26505, USA; ^4^Evidera, Lexington, MA 02420, USA; ^5^Sanofi U.S., Inc., Bridgewater, NJ 08807, USA

## Abstract

*Objective.* To examine the association between metformin use and cancer stage at diagnosis among elderly men with preexisting diabetes mellitus and incident prostate cancer.* Methods*. This study used a population-based observational cohort of elderly men (≥66 years) with preexisting diabetes and incident prostate cancer between 2008 and 2009 (*N* = 2,652). Cancer stage at diagnosis (localized versus advanced) was based on the American Joint Cancer Committee classification. Metformin use and other independent variables were measured during the one year before cancer diagnosis. Logistic regressions with inverse probability treatment weights were used to control for the observed selection bias.* Results*. A significantly lower percentage of metformin users were diagnosed with advanced prostate cancer as compared to nonusers (4.7% versus 6.7%, *p* < 0.03). After adjusting for the observed selection bias and other independent variables, metformin use was associated with a 32% reduction in the risk of advanced prostate cancer (adjusted odds ratio, AOR: 0.68, 95% confidence interval, CI: 0.48, 0.97).* Conclusions*. This is the first epidemiological study to support the role of metformin in reducing the risk of advanced prostate cancer. Randomized clinical trials are needed to confirm the causal link between metformin use and prostate cancer diagnosis stage.

## 1. Introduction

Individuals with diabetes, specifically those with type 2 diabetes mellitus (T2DM) have a higher risk for many cancers such as the breast, colon and rectum, endometrium, liver, and pancreatic cancers as compared to those without diabetes [1] due to biological mechanisms and shared risk factors [2–4]. In preclinical studies, T2DM has been found to be associated with increased levels of plasma insulin, insulin resistance, and hyperglycemia, which may have a direct effect on the growth of tumors [5, 6] leading to the development of many types of cancers [1].

In the case of prostate cancer, an inverse relationship between diabetes and cancer risk has been observed [3]. However, among men who developed prostate cancer, diabetes was associated with an advanced stage of cancer diagnosis [7, 8]. Three population-based studies in the United States (US) reported that the presence of diabetes was associated with an increased risk for advanced prostate cancer measured either by stage or tumor grade. In case-control studies and cohort studies, men with diabetes were less likely to be diagnosed with localized stage of prostate cancer. The risk ratio (RR) was 0.70, 95% CI = 0.56–0.86 for case-control studies, and the RR was 0.72, 95% CI 0.67, 0.77 for cohort studies [7]. Although the exact biological mechanisms for the link between diabetes and prostate cancer are not known, it is speculated that men with diabetes have low levels of androgen, which may be associated with advanced stage of cancer at diagnosis [7, 8].

The main modality of treatment for diabetes is pharmacotherapy with antidiabetes drugs such as metformin, thiazolidinediones, sulfonylureas, and insulin. All classes of antidiabetes drugs may indirectly affect the risk of prostate cancer by controlling hyperglycemia. Of special interest is the use of metformin for diabetes management because of its unique actions on insulin resistance and hyperinsulinemia [9] as well as its anticancer properties [10–12]. A systemic review found that metformin use reduced the risk of prostate cancer among men with diabetes [13] perhaps by regulating adenosine monophosphate-activated protein kinase (AMPK) pathways [10] and mammalian target of rapamycin (mTOR) [12]. Based on preclinical evidence, one can speculate that metformin may also reduce the risk of advanced prostate cancer among men with diabetes and incident prostate cancer [14]. This relationship between metformin and advanced prostate cancer diagnosis was explored by one population-based study from Canada [15]. The study investigators used a cohort of 119,315 men with diabetes and measured cancer stage using tumor grade. After adjusting for other risk factors, the investigators concluded that metformin use was not associated with the advanced form of cancer diagnosis [15]. The investigators of the study did not control for severity of diabetes, which may have affected the findings. Controlling for diabetes severity is important because individuals with severe diabetes have micro- and macrovascular complications and may have adverse pathological profiles [16]. Therefore, diabetes severity may alter the relationship between metformin use and cancer stage at diagnosis. Furthermore, the study did not control for the observed selection bias between metformin users and nonusers; such bias may lead to misleading findings on the association between metformin use and cancer stage at diagnosis. Thus, there is a need for population-based studies to examine the relationship between metformin use and cancer stage at diagnosis that control for a comprehensive set of risk factors and the observed selection bias between metformin users and nonusers.

Therefore, the current study is conducted to investigate the association between metformin use and cancer stage at diagnosis among elderly fee-for-service Medicare beneficiaries with T2DM and incident prostate cancer.

## 2. Methods

### 2.1. Study Design

**Figure 1:**
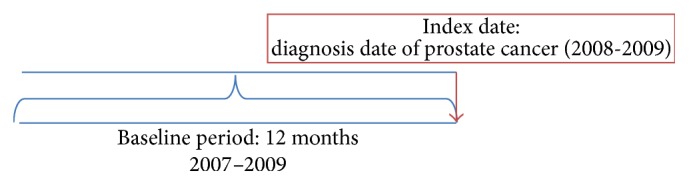
Schematic presentation of study design to examine the relationship between metformin use and cancer stage at diagnosis.

A cohort study design was adopted with a baseline and an index date as depicted in Figure 1. The index date was defined as the date of diagnosis of prostate cancer. The baseline period consisted of 12 months before the index date. Diabetes, metformin use, and other independent variables were identified during the baseline period. The types of initial cancer treatment were identified during the follow-up period.

### 2.2. Data Source

Data were derived from the SEER-Medicare linked database. The SEER data comprised 18 population-based cancer registries having precise and accurate information on all newly diagnosed cancer cases since 1973. At present, the data consisted of a total of 1,847,363 cases of all cancers and 340,769 cases of prostate cancer among the elderly population whose age was at least 65 at the time of diagnosis of cancer presenting. With 98% ascertainment of cases with the medical records and the highest level of certification of data quality from the North American Association of Central Cancer Registries, the SEER data are considered to be the most comprehensive and high-quality population-based data on cancer incidence and their treatment and outcomes. The Patient Entitlement and Diagnosis Summary File (PEDSF) provides information on the cancer diagnosis up to ten cancers, types of cancers, cancer stage, individual's demographic attributes, marital status, and tumor characteristics at the time of cancer diagnosis. The Medicare is the primary health insurer for 97% of the US population aged 65 years and older [17]. A total of 93% of men aged 65 years and older in SEER have been linked to Medicare population enrollment records [18]. The Medicare part of the database is comprised of the Medicare Provider Analysis and Review (MEDPAR) files, the carrier claims (old name, physician/supplier (NCH)), outpatient (OUTPT), and Part D Event (PDE). The MEDPAR and outpatient file include Medicare Part A claims records from any short term or long term hospital or skilled nursing facility stay from each calendar year while the outpatient file had Part B claims with outpatient visits. Each record represents an episode of hospital stay and has up to 10 diagnoses according to the* International Coding of Diseases, 9th Edition-Code Modification (ICD-9-CM)* and 10* ICD-9-CM* procedures during each stay, on the day of admission, and on the day of discharge. The carrier file represents billing records from physicians and noninstitutional providers and has procedure codes according tothe Health Care Procedure Classification Code (HCPCS) and the Common Procedural Terminology, 4th Edition (CPT-4) and ICD-9-CM procedure codes with service dates [19]. Medicare introduced optional/volunteer Part D plans in 2006 that cover prescription drugs benefit through enrollment in a Medicare advantage prescription drug plan (MA-PD) or a stand-alone drug plan (PDP). Almost half of the Medicare Parts A and B enrollee were also enrolled in Part D plans [20]. We utilized the data of the Medicare Part D Event (PDE) file in addition to PEDSF, MEDPAR, OUTPT, and NCH for the years 2007 to 2010. We examined prostate cancer cases diagnosed between 2008 and 2009 so that we could study their medication status in a year prior to diagnosis of cancer.

### 2.3. Study Cohort

**Figure 2:**
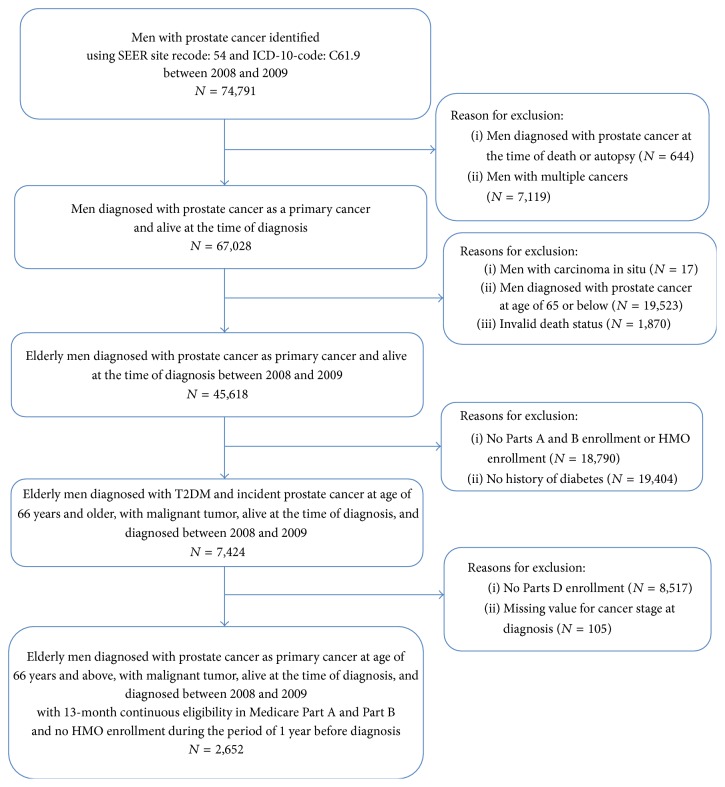
Study cohort development flow diagram for study population of elderly Medicare beneficiaries diagnosed with prostate cancer and diabetes.

The study cohort comprised 45,618 men with incident prostate cancer diagnosed between 2008 and 2009. We excluded 42,966 cases for the following reasons: those diagnosed with prostate cancer during the autopsy; those who had multiple cancers; those who had carcinoma in situ; those aged 65 years and younger; those who died during the study period; those enrolled in Medicare Health Maintenance Organizations; those not continuously enrolled in Medicare Parts A, B, and D during the study period, and those missing cancer stage at diagnosis. The details on the study cohort selection process are provided in Figure 2.

After all the exclusions, the final study cohort consisted of 2,652 elderly men with type 2 diabetes mellitus (T2DM) and incident prostate cancer. To note, every year nearly half of enrolled Medicare beneficiaries in Parts A and B are also enrolled in Medicare Part D since 2006. Therefore, our study population with prostate cancer and T2DM reduced to 7,424 enrolled in Medicare Parts A and B to 2,652 with enrollment Part D. To reduce the selection bias, we compared the characteristics of elderly men with T2DM and incident prostate cancer enrolled in Medicare Parts A and B to those enrolled in Parts A, B, and D as shown in the Appendix. These two sets of population shared similar characteristics.

### 2.4. Key Dependent Variable

#### 2.4.1. Cancer Stage at Diagnosis

The American Joint Committee on Cancer (AJCC) Tumor-Node-Metastases (TNM) classification was used to identify the stage of prostate cancer from the PEDSF file. Based on the AJCC-TNM systems, men were classified as having localized cancer stage if they had T1 or T2 clinical stage with no regional lymph node (NX-N0) involvement and absence of any distant metastasis (M0) [21]. Men were classified as having advanced cancer if they were diagnosed with T3 or T4 clinical stage with or without regional lymph node (N1) or distant metastasis (M1).

### 2.5. Key Independent Variable

#### 2.5.1. Metformin Use

Metformin use was identified using the Medicare Part D files. Metformin prescriptions were identified using the national drug codes (NDCs). Men with at least one prescription for metformin during the baseline period were considered as metformin users and men without any prescriptions for metformin were considered as nonusers.

### 2.6. Other Independent Variables

#### 2.6.1. Conceptual Framework

We utilized the Anderson Healthcare Behavior and Utilizations Model (ABM) model [22] to classify the potential independent factors associated with advanced prostate cancer.

*Predisposing Characteristics.* Predisposing characteristics consisted of age at diagnosis, race, and marital status. These were identified from the PEDSF. Age at the time of diagnosis was categorized into two groups (66 to 74 years, ≥75 years). Race/ethnicity was categorized into four groups: White, African American, Latino, and others. Marital status was categorized into four groups: married, divorced/separated, unmarried, and others.

*Enabling Characteristics*. Enabling characteristics were as follows: education, income, prostate specific antigen test, and visits to primary care physicians. Median income and median education at the census tract of residence were derived from the PEDSF. Income and education were measured by quartiles. The receipt of PSA test was identified using the following HCPCS codes: 84152, 84153, 84154, and G0103 using the Medicare carrier files during the baseline period. The presence or absence of primary care visits during the baseline was identified using the provider specialty codes [23].

*Need Characteristics.* The presence of T2DM was identified using at least one inpatient visit or two or more physician visits with a primary or a secondary diagnosis codes for T2DM (*ICD-9-CM codes: 250.x0 or 250.x2*) during the baseline period. The severity of diabetes, oral antidiabetic medication use, insulin use, statins use, and corticosteroid use were considered as need factors. The Diabetes Complications Severity Index (DCSI) was calculated using the modified algorithm by Chang et al. [24]. The DSCI is based on seven categories: retinopathy, nephropathy, neuropathy, cerebrovascular, cardiovascular, peripheral vascular disease, and metabolic conditions. Based on the severity of particular types of complications, a score of 1 or 2 was assigned to each of the seven categories, with a total DCSI score ranging from 0 to 13. The DCSI scores were grouped into quartiles. Details of the ICD-9-CM codes and the scoring algorithm are provided in the Appendix. The use of oral antidiabetic medications, insulin, statins, and corticosteroids was identified using the NDCs recorded in the Medicare Part D files during the baseline period.

*External Environment Characteristics*. The SEER data has 18 registries/regions which were categorized into four regions: (1) Northeast, with two registries of Connecticut and New Jersey; (2) South, with five registries of Kentucky, Louisiana, Atlanta, Rural Georgia, and Greater Georgia; (3) North-central, with two registries of Detroit and Iowa; and (4) West, with Hawaii, New Mexico, Seattle-Puget Sound, Utah, San Francisco-Oakland, San Jose-Monterey, Los Angeles, Greater California, Arizona, Alaska, and Cherokee Nation. The Area Healthcare Resource Use Files (AHRF) were linked to identify the number of radiation oncology units, and urology units at the county-level with cancer cases and quartiles of total radiation oncology and urology units were created for each case [25].

### 2.7. Statistical Analyses

Significant group differences in the study population characteristics by metformin use were examined with chi-square tests. A binary logistic regression was used to determine the associations between predisposing, enabling, need, and external environment characteristics and metformin use. C-statistics and area under the curve were used to assess the model fit. The logistic regression was used to derive inverse probability treatment weights (IPTWs) and these standardized IPTWs were used to control for the observed selection bias in regressions on the cancer stage.

Significant unadjusted associations between metformin use and cancer stage at diagnosis were examined with chi-square tests. The IPTW-adjusted multivariable logistic regressions were used to analyze the relationship between metformin use and cancer stage at diagnosis. As the odds ratios and relative risk are approximately similar for the events with low prevalence, such as advanced prostate cancer (≤10%) [26], these terms risk ratio or odds ratio of advanced prostate cancer were used interchangeably. All statistical analyses were carried out using* Statistical Analysis System* (SAS) version 9.4 (SAS Institute Inc., Cary, NC).

## 3. Results

### 3.1. Description of the Study Cohort

The study cohort consisted of 2,652 elderly men with preexisting T2DM and incident prostate cancer between 2008 and 2009.  Table 1 represents the characteristics of the study cohort. An overwhelming majority of men were Whites (92.3); 59.7% were diagnosed with prostate cancer between the ages of 66 and 74 years; 58.6% were married; and 46.2% resided in the Western region of the US. Nearly three-quarters (70.0%) of study cohort had a primary care visit. An overwhelming majority (91.1%) had PSA test during the baseline period.

### 3.2. Description of the Study Cohort by Metformin Use

Table 1 also summarizes the characteristics of the study cohort by metformin use. Overall, 35.6% of the study cohort had at least one prescription of metformin during the baseline period. Significant differences in predisposing, enabling, need, and external environment factors by metformin use were observed.

Table 2 describes the adjusted odds ratios (AOR) and 95% CI for the metformin use among elderly men with diabetes and prostate cancer. Elderly men aged 66 to 74 years as compared to those aged 75 years and older (AOR: 1.31, 95% CI: 1.10, 1.56), Latinos as compared to Whites (AOR: 1.62, 95% CI: 1.12, 2.34), those who received insulin as compared to no insulin (AOR: 2.95, 95% CI: 1.94, 4.49), and those who received statins as compared to no statins (AOR: 1.94, 95% CI: 1.63, 2.31) were more likely to receive metformin. Whereas, elderly men with a severe DCSI score (4 to 13) were more likely to receive metformin as compared to those with zero or one DCSCI score (AOR: 0.65, 95% CI: 0.51, 0.84).

### 3.3. Metformin and Cancer Stage at Diagnosis

Table 3 describes the relationship between metformin use and cancer stage at diagnosis among elderly men with prostate cancer and diabetes. Overall, 93.7% of the study population was diagnosed with localized prostate cancer; 6.3% was diagnosed with advanced prostate cancer. A significantly lower percentage of metformin users were diagnosed with advanced prostate cancer as compared to nonusers (4.7% versus 6.7%, *p* < 0.03).

Table 3 also reports unadjusted odds ratios (OR) and adjusted ORs (AOR) from IPTW logistic regressions for the advanced prostate cancer among elderly men with T2DM and prostate cancer. In unadjusted logistic regression, we observed the association between metformin and risk of advanced prostate cancer at diagnosis (OR: 0.69, 95% CI: 0.49, 0.95). After adjusting for predisposing, enabling, need, and external environment factors among elderly men with prostate cancer and diabetes, metformin use was significantly associated with a reduction in the risk of advanced prostate cancer (AOR: 0.68, 95% CI: 0.48, 0.97).

## 4. Discussion

The current study is the first largest population-based study to examine whether the risk of advanced prostate cancer diagnosis is reduced with metformin use among elderly men with preexisting T2DM and incident prostate cancer in the US. Characteristics of metformin users were consistent with literature. We observed that a higher percentage of men living in the Western region of the united states were prescribed metformin as compared to those living in other regions. Census-track level income was also associated with metformin use. These study findings are consistent with one published study on geographical disparities in antidiabetes medications. Regional disparities in metformin use can reflect practice patterns in regions [26].

We found that the risk for advanced stage cancer diagnosis is reduced with metformin use among elderly men with T2DM and incident prostate cancer after controlling for the observed section bias between metformin users and nonusers and other independent variables. The current study addressed the limitations of the single population-based examination of metformin use and the cancer stage by incorporating a validated diabetes severity complications index and controlling for the observed selection bias. The current study findings are consistent with the preclinical evidence on the role of metformin in prevention of advanced prostate cancer [10, 27]. Further, it should be noted that neither oral antidiabetic medications (sulfonylurea and thiazolidinedione) nor insulin use was associated with advanced prostate cancer (*data not shown in tabular format*). Therefore, it is plausible that metformin may act via insulin-independent pathways to reduce the risk of advanced prostate cancer. If the findings of the current study are confirmed by other population-based studies, randomized clinical trials can be conducted to establish the causal link between metformin use and risk of advanced prostate cancer diagnosis.

It should be noted that the current study controlled for the observed selection bias because metformin users and nonusers were significantly different with respect to their predisposing, enabling, need, and external environment characteristics. Without adjustments for the observed selection bias, there was not a statistically significant difference in the cancer stage between metformin users and nonusers. Therefore, accounting for the observed selection bias is important in establishing an association between metformin use and the reduction in the risk of advanced prostate cancer diagnosis.

The current study has a number of strengths. The large cohort size and high-quality data on the clinical and pathological features of cancer at the time of diagnosis enabled us to examine not only the association between metformin use and the cancer stage diagnosis but also the initial choice of cancer treatment [18]. Furthermore, the inclusion of variable on severity of diabetes using a validated method enabled controlling and relating the effect of severity of disease on the risk of advanced prostate cancer at diagnosis and receipt of initial cancer treatment.

The current study has some limitations as well. The prescription claims for metformin and other drugs were used. Filling the prescriptions cannot be equated to the actual use of these drugs. We made an attempt to overcome this issue to some extent via measuring the one-year adherence to medication, and a lower proportion of those with adherence to metformin and nonadherent metformin had advanced prostate cancer as compared to nonusers. Due to lack of sufficient sample size, we did not present the results in tabular format. However, the direction of our findings is consistent with the previous studies suggesting no difference in the ever metformin users and adherent metformin users on grade of prostate cancer [15]. Secondly, we focused on elderly men with prostate cancer because two-thirds of prostate cancers cases are diagnosed among the elderly men aged 65 years and older [28]; however, the study population consisted of elderly men with T2DM residing in SEER-regions and enrolled in fee-for-services Medicare Parts A, B, and D plans; therefore, one cannot generalize the study finding to younger men or all Medicare beneficiaries with incident prostate cancer in the US. Information on many important prognostic factors such as body-mass index and smoking could not be adjusted; these factors may be associated with an increased risk of advanced prostate cancer diagnosis. Duration of metformin use could not be adjusted due to data limitations. Future studies need to examine whether a greater duration of metformin use is associated with a decrease in the risk of advanced prostate cancer at diagnosis. As the study population was restricted to elderly men with T2DM, the current study findings cannot be generalized to men without T2DM and incident prostate cancer.

## 5. Conclusions

Metformin use was associated with a statistically significant reduction in the risk advanced prostate cancer diagnosis among elderly men with T2DM and incident prostate cancer. The current study findings highlight the need for additional studies in this area. Other population-based studies need to be conducted to confirm the study findings. If confirmed, randomized controlled trials can be carried out to examine the causal link between metformin use and the risk of advanced prostate cancer diagnosis.

## Figures and Tables

**Table 1:** Characteristics of the study cohort elderly Medicare beneficiaries with diabetes and incident prostate cancer by metformin use SEER-Medicare linked database, 2007–2010.

All	Overall		Metformin users		Non-metformin-user		Sig.
	*N*	%	*N*	%	*N*	%	
	2,652	100	948	35.7	1,704	64.3	
*Predisposing characteristics*							

*Age at diagnosis, in years*							*∗∗∗*
66–74	1,579	59.5	609	64.2	970	56.9	
75+	1,073	40.5	339	35.8	734	43.1	
*Race/ethnicity*							*∗*
Whites	1,914	72.2	680	71.7	1,234	72.4	
African American	323	12.2	108	11.4	215	12.6	
Hispanic/Latino	150	5.7	70	7.4	80	4.7	
Others	265	10.0	90	9.5	175	10.3	
*Marital status*							
Unmarried	222	8.4	73	7.7	149	8.7	
Married	1,550	58.4	581	61.3	969	56.9	
Divorced/separated	393	14.8	136	14.3	257	15.1	
Others	487	18.4	158	16.7	329	19.3	

*Enabling characteristics*							

*Quartile of median census of 2000 income*							*∗*
$7–$34,522	664	25.0	265	28.0	399	23.4	
$34,523–46,224	664	25.0	243	25.6	421	24.7	
$46,229–62,764	664	25.0	229	24.2	435	25.5	
$62,767–200,008	660	24.9	211	22.3	449	26.3	
*Quartile of median census of 2000 education*							
0–8.52	666	25.1	222	23.4	444	26.1	
8.53–15.16	655	24.7	229	24.2	426	25.0	
15.17–26.09	664	25.0	248	26.2	416	24.4	
26.1–100	667	25.2	249	26.3	418	24.5	
*PSA screening*							
Yes	2,415	91.1	872	92.0	1,543	90.6	
No	237	8.9	76	8.0	161	9.4	
*Visit to a PCP*							
Yes	1,858	70.1	672	70.9	1,186	69.6	
No	794	29.9	276	29.1	518	30.4	
*Statin use*							*∗∗∗*
Yes	1,576	59.4	656	69.2	920	54.0	
No	1,076	40.6	292	30.8	784	46.0	
*Insulin*							*∗∗∗*
Yes	102	3.8	62	6.5	40	2.3	
No	2,550	96.2	886	93.5	1,664	97.7	

*External environment characteristics*							

*SEER-regions*							*∗∗*
Northeast	487	18.4	146	15.4	341	20.0	
South	570	21.5	223	23.5	347	20.4	
North-central	351	13.2	112	11.8	239	14.0	
West	1,244	46.9	467	49.3	777	45.6	

*Need characteristics*							

*DCSI quartile*							*∗∗*
0 to 0	846	31.9	305	32.2	541	31.7	
1 to 1	476	17.9	195	20.6	281	16.5	
2 to 3	811	30.6	297	31.3	514	30.2	
4 to 13	519	19.6	151	15.9	368	21.6	

Notes: based on the data of 2,652 elderly men aged 66 years and older diagnosed with prostate cancer between 2008 and 2009 using a surveillance, epidemiology, and end-results- (SEER-) Medicare linked Part D data. Significant group differences by metformin use are based on chi-square tests. % represented in the column is row percentages.

DCSI: diabetes complication severity index; PCP: primary care physician; PSA: prostate specific antigen; Sig.: level of significance. ^*∗∗∗*^ *p* < 0.001; ^*∗∗*^0.001 ≤ *p* < 0.01; ^*∗*^0.01 ≤ *p* < 0.05.

**Table 2:** Adjusted odds ratios and 95% confidence intervals from logistic regressions on metformin use among elderly Medicare beneficiaries with diabetes incident prostate cancer surveillance, epidemiology, and end-results- (SEER-) Medicare linked data, 2007–2010.

	AOR	95% CI	Sig.
*Predisposing characteristics*			

*Age at diagnosis*			
66–74 years	1.31	[1.10, 1.56]	*∗∗*
75 years or above	Ref.		
*Race/ethnicity*			
Whites	Ref.		
African American	0.91	[0.69, 1.21]	
Latino	1.62	[1.12, 2.34]	*∗∗*
Others	0.80	[0.59, 1.10]	
*Marital status*			
Married	Ref.		
Unmarried	0.81	[0.59, 1.11]	
Divorced/separated	0.94	[0.74, 1.20]	
Others	0.83	[0.66, 1.04]	

*Enabling characteristics*			

*Quartile of median census of 2000 income*			
$7–$34,522	1.72	[1.20, 2.46]	*∗∗*
$34,523–46,224	1.34	[0.97, 1.84]	
$46,229–62,764	1.18	[0.89, 1.56]	
$62,767–200,008	Ref.		
*Quartile of median census of 2000 education*			
0–8.52	1.24	[0.87, 1.77]	
8.53–15.16	1.21	[0.90, 1.62]	
15.17–26.09	1.18	[0.92, 1.51]	
26.1–100	Ref		
*Visit to a PCP*			
Yes	1.12	[0.92, 1.35]	
No	Ref.		
*Statin use*			
Yes	1.94	[1.63, 2.31]	*∗∗∗*
No	Ref		
*Insulin*			
Yes	2.95	[1.94, 4.49]	*∗∗∗*
No	Ref.		

*Need factors*			

*DCSI quartile*			
0 to 1	Ref.		
2 to 2	1.13	[0.89,1.44]	
3 to 3	0.98	[0.79, 1.21]	
4 to 13	0.65	[0.51, 0.84]	*∗∗∗*

*External environment characteristics*			

*SEER-regions*			
Northeast	Ref.		
South	1.40	[1.06, 1.85]	*∗*
North-central	1.07	[0.78, 1.47]	
West	1.37	[1.08, 1.75]	*∗*

Notes: based on the data of 2,652 elderly men aged 66 years and older diagnosed with prostate cancer between 2008 and 2009 using a surveillance, epidemiology, and end-results- (SEER-) Medicare linked Part D data. Significant group differences are based on log-likelihood test for metformin use.

DCSI: diabetes complication severity index; PCP: primary care physician; Ref.: reference group; Sig.: level of significance; ^*∗∗∗*^ *p* < 0.001; ^*∗∗*^0.001 ≤ *p* < 0.01; ^*∗*^0.01 ≤ *p* < 0.05.

**(a):** 

	Localized		Advance		Sig.
Overall	*N*	Weighted%	*N*	Weighted%	
	2,493	93.7	159	6.3	
*Metformin use*					*∗*
Yes	902	95.3	46	4.7	
No	1,591	93.3	113	6.7	

**(b):** 

Advanced stage at diagnosis IPTW					

*Unadjusted odds ratios and 95% confidence intervals*					
	OR	95% CI	Sig.		

*Metformin*					
Yes	0.69	[0.49, 0.95]	*∗*		
No	Ref.				

*Adjusted odds ratios and 95% confidence intervals*					
	AOR	95% CI	Sig.		

*Metformin*					
Yes	0.68	[0.48, 0.97]	*∗*		
No	Ref.				

Notes: based on the data of 2,652 elderly men aged 66 years and older diagnosed with prostate cancer between 2008 and 2009 using a surveillance, epidemiology, and end-results- (SEER-) Medicare linked data. % is weighted percentage for IPTW. Significant differences are based on the log-likelihood test using a logistic regression with IPTW weights. Adjusted model controlled for predisposing, enabling, need, and external environment related factors.

IPTW: inverse probabilities treatment weights; PSA: prostate specific antigen level; PCP: primary care physician visit; Sig.: level of significance; ^*∗∗∗*^ *p* < 0.001; ^*∗∗*^0.001 ≤ *p* < 0.01; ^*∗*^0.01 ≤ *p* < 0.05.

**Table 4:** Codes and algorithms to identify diabetes complication severity index (DCSI) developed by Young et al. and modified by Chang et al.

Complications	ICD-9-CM code	DCSI score
*Retinopathy*		
Diabetic ophthalmologic disease	250.5x	1
Background retinopathy	362.01	1
Other retinopathies	362.1	1
Retinal edema	362.83	1
CSME	362.53	1
Other retinal disorders	362.81, 362.82	1
Proliferative retinopathy	362.02	2
Retinal detachment	361.xx	2
Blindness	369.xx.00–0.99	2
Vitreous hemorrhage	379.23	2

*Nephropathy*		
Diabetic nephropathy	250.4	1
Acute glomerulonephritis	580	1
Nephrotic syndrome	581	1
Hypertension, nephrosis	581.81	1
Chronic glomerulonephritis	582	1
Nephritis/nephropathy	583	1
Chronic renal failure	585	
Renal failure NOS	586	
Renal insufficiency	593.9	

*Neuropathy*		
Diabetic nephropathy	356.9, 250.6	1
Amyotrophy	358.1	1
Cranial nerve palsy	951.0, 951.1, 951.3	1
Mononeuropathy	354.0–355.9	1
Charcot's arthropathy	713.5	1
Polyneuropathy	357.2	1

*Cerebrovascular*		
TIA	435	1
Stroke	431, 433, 434, 436	2

*Cardiovascular*		
Atherosclerosis	440.xx	1
Other IHD	411	1
Angina pectoris	413	1
Other chronic IHD	414	1
Myocardial infarction	410	2
Ventricular fibrillation, arrest	427.1, 427.3	2
Atrial fibrillation, arrest	427.4, 427.5	2
Other ASCVD	429.2	1
Old myocardial infarction	412	2
Heart failure	428	2
Atherosclerosis, severe	440.23, 440.24	2
Aortic aneurysm/dissection	441	2

*Peripheral vascular disease*		
Diabetic PVD	250.7	1
Other aneurysms, LE	442.3	1
PVD	443.81, 443.9	1
Foot wound + complication	892.1	1
Claudication, intermittent	443.9	1
Embolism/thrombosis (LE)	444.22	2
Gangrene	785.4	2
Gas gangrene	0.4	2
Ulcer of lower limbs	707.1	2

*Metabolic*		
Ketoacidosis	250.1	2
Hyperosmolar	250.2	2
Other comas	250.3	1

Note: the table is adapted from the previous algorithm defined by Young et al. and modified by Change et al. to identify the severity of diabetes using claims database. Severity index was based on a scale ranging from 0 to 2 for each complication as follows: 0 = no abnormality, 1 = some abnormality, and 2 = severe abnormality.

ASCVD, atherosclerotic cardiovascular disease; CSME, cystoid macular edema/degeneration; DCSI, diabetes complications severity index; IHD, ischemic heart disease; ICD-9-CM, international classification of diseases, ninth revision, clinical modification; LE, lower extremity; NOS, not otherwise specified; PVD, peripheral vascular disease; TIA, transient ischemic attack.

**Table 5:** Baseline characteristics of elderly men with prostate cancer with T2DM overall and enrolled in Part D program SEER-Medicare linked database, 2008-2009.

All	DM Part D (*N* = 2,652)	DM overall (*N* = 7,424)
	%	%
*Age at diagnosis, in years*		
66–74	59.5	59.4
75+	40.5	40.6

*Race/ethnicity*		
Whites	72.2	75.3
African American	12.2	14.5
Hispanic/Latino	5.7	3.0
Others	10.0	7.1

*Marital status*		
Unmarried	8.4	6.6
Married	58.4	63.2
Divorced/separated	14.8	14.0
Others	18.4	16.2

*Quartile of median census of 2000 income*		
$7–$34,522	25.0	24.9
$34,523–46,224	25.0	25.1
$46,229–62,764	25.0	25.0
$62,767–200,008	24.9	25.0

*Quartile of median census of 2000 education*		
0–8.52	25.1	25.0
8.53–15.16	24.7	25.0
15.17–26.09	25.0	25.1
26.1–100	25.2	25.0

*Visit to a PCP*		
Yes	70.1	65.0
No	29.9	35.0

*PSA screening in past year*		
Yes	91.1	88.4
No	8.9	11.6

*SEER-regions*		
Northeast	18.4	19.3
South	21.5	24.9
North-central	13.2	11.8
West	46.9	44.0

*Quartile of radiation oncology*		
0 to 1	26.1	26.1
2 to 6	24.7	24.7
7 to 22	25.3	25.3
23 to 147	23.9	23.9

*Quartile of urology centers*		
0 to 3	24.5	24.5
4 to 16	24.7	24.7
17 to 44	26.2	26.2
45 to 343	24.5	24.5

*Year of diagnosis*		
2008	50.9	51.6
2009	49.1	48.4

## Appendix

See Tables 4 and 5.
